# Trace Metals in Nectar of Important Urban Pollinator Forage Plants: A Direct Exposure Risk to Pollinators and Nectar‐Feeding Animals in Cities

**DOI:** 10.1002/ece3.71238

**Published:** 2025-04-15

**Authors:** Sarah B. Scott, Mary M. Gardiner

**Affiliations:** ^1^ Department of Entomology The Ohio State University Columbus Ohio USA

**Keywords:** metal exposure routes, pollinator conservation, pollinator foraging, trace metal contamination, urban greenspaces

## Abstract

Pollinators are exposed to metals while foraging in the landscape and accumulate detectable concentrations of trace metals within their bodies, although major exposure routes remain unclear. As nectar is the main source of food for pollinators, we analyzed trace metal content within floral rewards to identify if nectar contained detectable metals and may serve as an oral exposure route. Nectar from flowering plant species growing within vacant lots in the city of Cleveland, OH, USA was extracted using a centrifuge and tested for the metals arsenic, cadmium, chromium, and lead using ICP‐MS. We collected volunteer flower species that are common pollinator forage plants. Nectar metal content varied by plant and metal species, but not by location. Nectar arsenic concentrations ranged from 0 to 8.44 μg/L, cadmium from 0 to 32.99 μg/L, chromium from 0 to 45.69 μg/L, and lead from 0 to 135.31 μg/L. The presence of these soil contaminants in nectar indicates that the uptake and concentration of metals within nectar resources is likely a major route of metal exposure for pollinators and nectar‐feeding animals.

## Graphical abstract

Pollinators accumulate trace metals while foraging, but theimportance of nectar as an exposure route was unclear. This study analyzed nectar from urban flowers in Cleveland, OH, finding that metal content varied by plant and metal type, though not by plant location. Results highlight that metal exposure risks depend on specific plant and soil interactions, not just soil metal levels.

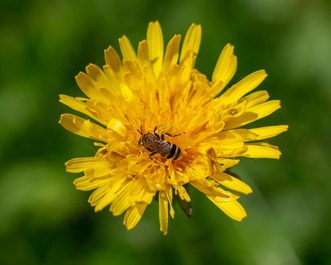

## 1 Introduction

Global pollinator declines have prompted a call to action to conserve and establish new pollinator habitat patches to aid in counteracting population losses (Brown et al. 2016). Cities can support species‐rich communities of wild bees (McFrederick and LeBuhn 2006; Hall et al. 2017; Senapathi et al. 2017; Theodorou et al. 2020). However, environmental stressors including habitat fragmentation, elevated temperatures, pollution, and pesticide use threaten urban habitat quality (McKinney 2002; Harrison and Winfree 2015; Aronson et al. 2016). In legacy cities, urban spaces such as vacant lots, brownfield sites, and road verges have received attention as potential locations to install pollinator habitat (Anderson and Minor 2017; Martins et al. 2017; Turo et al. 2021). However, these sites can be contaminated either from their history of land use or activities within the region, resulting in elevated trace metals, chemicals, and other pollutants wihtin the soil (Sharma et al. 2015a; Pecina et al. 2021).

Bees are exposed to metals during foraging, and this exposure can have negative impacts on bee health (Scott et al. 2023). For example, solitary bees near mining sites collect metals in brood provisions (Moroń et al. 2014), and bumble bees accumulate metals while foraging within urban areas (Sivakoff et al. 2020; Breidenbach et al. 2023). Bumble bee colonies exposed to environmentally realistic concentrations of metals can experience elevated brood mortality (Sivakoff et al. 2020; Scott et al. 2022), delayed time to oviposition in queens, and extended larval development time (Wu et al. 2025). The abundance and diversity of solitary bee populations have also been found to decline with increasing metal exposure (Moroń et al. 2012; Moroń et al. 2014). Exposure to metal concentrations can affect learning, memory, and many behaviors within honey bees (Rothman et al. 2019; Monchanin et al. 2021a, 2021b). Exposure of honey bees to metals can also result in elevated mortality (Ecology and Hydrology et al. 2016; Scott et al. 2024), reduction in flight ability (Gao et al. 2024), and reduced brain size and cognitive performance (Monchanin et al. 2024). Therefore, reducing the risk of metal exposure is critical for the success of urban pollinator conservation initiatives.

Identifying metal exposure routes is the first step towards mitigating the risks these pollutants pose to urban bees. It is likely that multiple sources contribute to overall metal body load (Scott et al. 2023). Although pesticide exposure routes are well studied (Kopit and Pitts‐Singer 2018; Gradish et al. 2019), metal exposure routes are less clearly defined, though they may share similar pathways with pesticides. As bees are reliant on floral resources as their exclusive source of food (Vaudo et al. 2015), the primary exposure route is likely direct contact with and consumption of any chemical (Belsky and Joshi 2020; Zioga et al. 2023) or metal (Xun et al. 2017) present within pollen and/or nectar resources.

**FIGURE 1:**
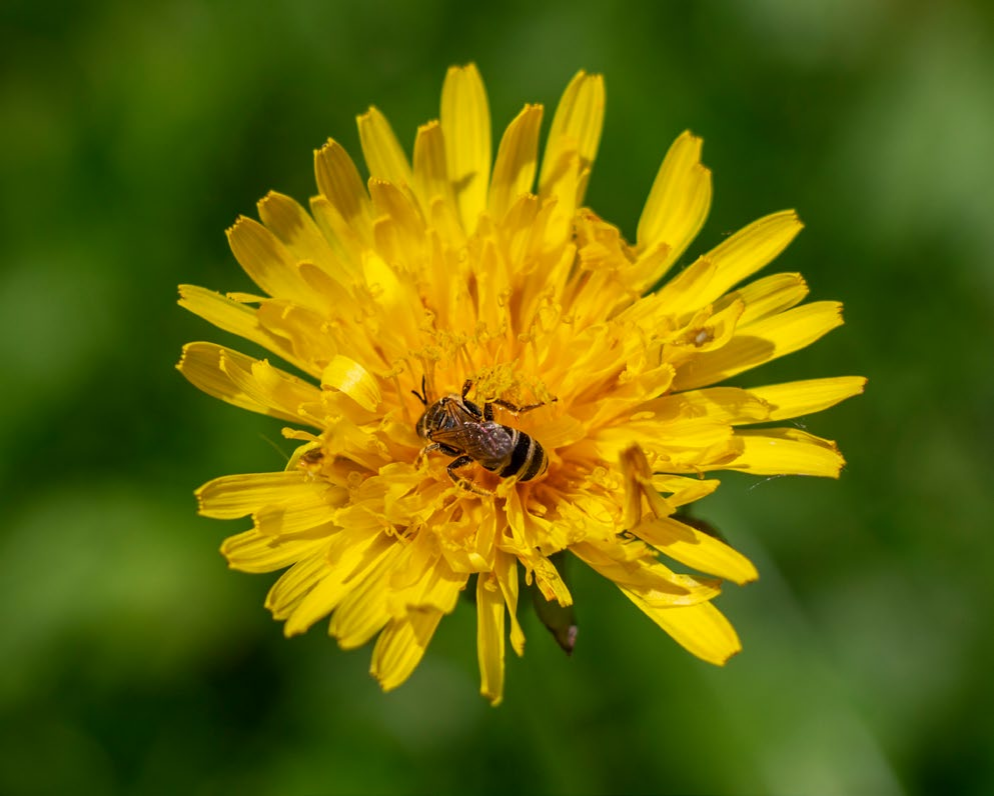
Volunteer plant species are important forage resources for native bees within urban areas.

Within vacant lots and other minimally managed urban spaces, spontaneous vegetation species including *Trifolium pratense* (Fabaceae), *Daucus carota* (Apiaceae), and *Chicorium intybus* (Asteraceae) serve as the primary forage for bees in many areas due to their prevalence, distribution, and continuous bloom time over the seasons, Figure 1 (Turo et al. 2021). These ruderal species are often found growing in sites that have elevated soil metals (Cheng et al. 2015; Sharma et al. 2015b). Plants vary widely in their ability to uptake metals from the soil into their biomass, as the bioavailability of metals depends on multiple biotic and abiotic factors, including abundance and solubility in soil, interaction with soil particles, sequestration and speciation, plant root systems, and the response of plants to seasonal cycles (Chojnacka et al. 2005). Certain plant species, known as hyperaccumulators, can uptake trace metals from the soil into their tissues (roots, shoots, and leaves) at concentrations that far exceed concentrations normally found in plant biomass (Rascio and Navari‐Izzo 2011). Globally, there are around 700 known species of hyperaccumulator plants (Reeves et al. 2018), many of which belong to families that serve as important forage for bees, including Asteraceae, Fabaceae, Brassicaceae, and Lamiaceae (Reeves et al. 2018; Kuppler et al. 2023). Currently, the status of metal uptake and transport into floral rewards is largely unknown for most species of urban spontaneous vegetation.

Legacy cities vacant land can support species‐rich communities of wild bees (Turo et al. 2021). These urban areas have experienced a substantial population loss, which results in an overabundance of abandoned homes and businesses that are eventually demolished, creating vacant land (Herrmann et al. 2016; Sampson et al. 2017). For example, Cleveland, Ohio, USA, has lost almost 60% of its peak population (Gibson 1998; Exner 2019) and maintains over 33,700 vacant lots (Western Reserve land Conservancy 2023). Our goal was to identify the ability of common pollinator forage plants growing in vacant lots with soil heavy metal contamination to act as a metal exposure route for bees through the uptake and concentration of metals into nectar resources. We collected data from a network of vacant lots located across eight neighborhoods within the city. Each vacant lot was mown monthly by the City of Cleveland Land Bank and contained a diversity of urban spontaneous vegetation. Flowers from these plant species were collected to test nectar for the presence of nonessential, trace metals cadmium, chromium, and lead, and metalloid arsenic (hereafter collectively referred to as metals).

## 2 Materials and Methods

### 2.1 Sample Collection

Flower samples were collected on June 27, 2022 from plants growing within vacant lots in eight different neighborhoods of Cleveland, OH, USA, a legacy city with a history of industrial activity including iron and steel production, oil refining, and automobile manufacturing. The neighborhoods sampled included: Buckeye, Central, Detroit Shoreway, Fairfax, Glenville, Hough, Slavic Village, and Tremont. Two vacant lots in each of the eight neighborhoods were sampled for flowers (*n* = 16). Each vacant lot consisted of an individual residential parcel measuring approximately 12 × 30 m, or 360 m^2^. The lots are managed by the City of Cleveland Land Bank and mown once monthly throughout the growing season (April–October).

Flower samples were collected from every flowering species growing within each vacant lot that had more than one flowering plant. Species collected included *Asclepias syriaca* (Apocynaceae) (*n* = 10), *Cichorium intybus* (Asteraceae) (*n* = 89), *Convolvulus arvensis* (Convolvulaceae) (*n* = 36), *Daucus carota* (Apiaceae) (*n* = 20), *Lotus corniculatus* (Fabaceae) (*n* = 113), *Securigera varia* (Fabaceae) (*n* = 87), *Taraxacum officinale* (Asteraceae) (*n* = 7), *Trifolium pratense* (Fabaceae) (*n* = 366), and *Trifolium repens* (Fabaceae) (*n* = 307). Flowering plants were located by walking and visually scanning the entire length and width of each lot. Flowers were sampled by hand by removing the flower head from the top of the stem and removing any sepals using a knife. Each species of flower collected within the same vacant lot was combined into one plastic bag for transportation and storage. Flower samples from multiple plants of the same species were combined into one sample per lot to reach the minimum required quantity of nectar for metal analysis (as per guidance by the Trace Element Research Laboratory, The Ohio State University, Columbus, OH, USA). Samples were stored in a −20°C freezer until processing.

### 2.2 Sap and Nectar Extraction

We developed a modified nectar collection method via centrifugation, adapted from Arnold and Michaels (2017). Flower samples were removed from the freezer and left to thaw for 1 h prior to processing. After removing any remaining sepals, stems, or leaves from the flower head, the heads were suspended in a fine mesh pouch, placed inside a 15 mL conical centrifuge tube, and secured at the top by twisting the mesh between the cap and the tube. Samples were centrifuged at 7000 RPM for 2 min. The flower material and mesh were discarded, and the resulting liquid (hereafter nectar) was stored at −20°C. Flower samples of each species collected from within the same vacant lot were grouped into a single sample. We acknowledge that the extracted fluid may include plant xylem (sap) rather than nectar alone. Additionally, some detected metals may originate from metal‐containing dust on floral surfaces incorporated into the samples.

### 2.3 Acid Digestion and ICP Analysis

Nectar samples were prepared for metal analysis at The Ohio State University (Columbus, OH, USA) in November 2022. Samples were digested in 2 mL of trace metals grade 2% w/v nitric acid (HNO_3_) in a water bath (90°C) for 30 min. Samples were diluted to 15 mL with deionized water after being removed from the water bath and cooled. Metal content was measured using inductively coupled plasma with mass spectroscopy (ICP‐MS) using a Perkin Elmer SCIEX ELAN DRCII and autosampler in the Trace Elemental Research Laboratory (OSU, Columbus, OH, USA). Nectar was tested for arsenic, cadmium, chromium, and lead concentrations. A 50‐ppb standard and blank sample were analyzed after every 10 test samples for QA/QC.

### 2.4 Statistical Analysis

All analyses were performed using R Studio (RStudio Team 2020). Differences in metal concentrations between species, site locations, different species within a site, and the same species between sites were compared using a Kruskal‐Wallis test as data were not normally distributed, as indicated by the Shapiro–wilk test of normality. Post hoc pairwise comparisons were performed using Dunn's test with a BH correction.

## 3 Results and Discussion

### 3.1 Nectar Metals by Plant Species

**FIGURE 2:**
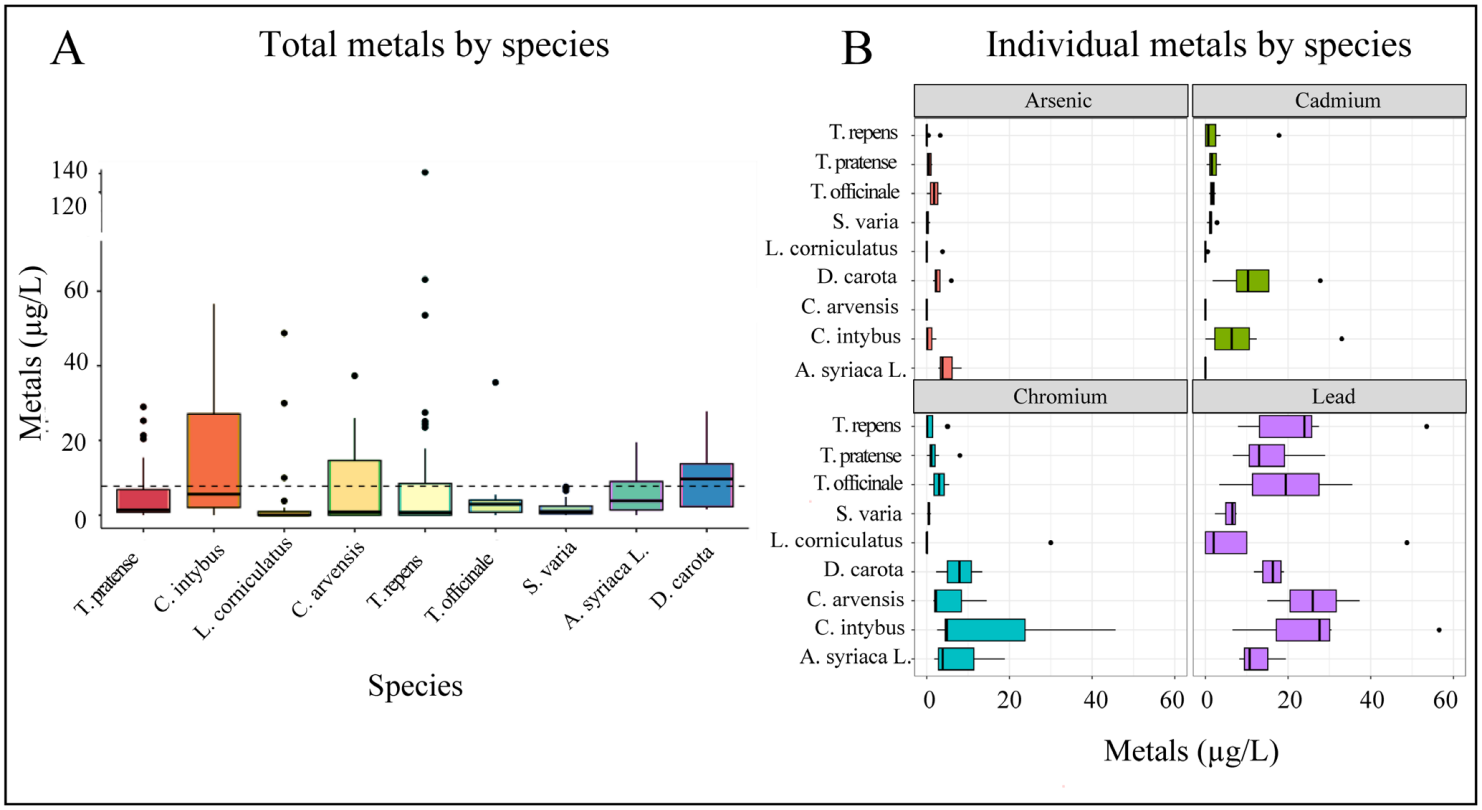
Nectar metal concentrations by plant species. (A) Total nectar metal (measured arsenic, cadmium, chromium, and lead) per species combined from all locations. The dotted line represents the average concentration of metals measured across all species. (B) Individual nectar metals per flower species. Each graph represents the total metals from all floral species from each site combined. *Cichorium intybus* accumulated significantly more nectar metals than *L. corniculatus* (*z* = 4.14, *p* < 0.05), *S. varia* (*z* = 2.71, *p* = 0.031), and *T. repens* (*z* = 2.72, *p* = 0.033). *Daucus carota* accumulated higher nectar metal concentrations than *C. arvensis* (*z* = 2.53, *p* = 0.045), *L. corniculatus* (*z* = 4.38, *p* < 0.05), *S. varia* (*z* = 3.13, *p* = 0.016), *T. pratense* (*z* = 2.47 *p* = 0.048), and *T. repens* (*z* = 3.14, *p* = 0.021). Finally, *Asclepias* spp. accumulated more metals within its nectar than *L. corniculatus* (*z* = 2.74, *p* = 0.037).

All plant species had nectar metal concentrations above the detectable limit (DL > 0.07 ppt). Trace metal uptake into nectar varied by plant species, and each plant species varied in the metals they accumulated (Figure 2, Table 1). Combined nectar metal concentrations of the four metals examined varied significantly by plant species (*χ* ^2^ (8) = 30.95, *p* < 0.01). *Cichorium intybus* accumulated the highest total nectar metal concentrations (60.7 ± 13.28 μg/L), followed by *Trifolium repens* (42.92 ± 12.50 μg/L), *Daucus carota* (39.26 ± 5.66 μg/L), and *Convolvulus arvensis* (32.22 ± 7.51 μg/L) (Table 1). Total nectar metal concentration by species followed the trend: *C. intybus* > *T. repens* > *D. carota* > *C. arvensis* > *A. syriaca* *L*. > *T. officinale* > *L. corniculatus* > *T. pratense* > *S. varia* . These plant species are vital forage for pollinators within legacy cities, often serving as the only available resource during specific seasons or in certain locations (Neil and Wu 2006). Within vacant lots, *Trifolium pratense* , *Daucus carota* , *Convolvulus arvensis* , and *Cichorium intybus* provide consistent and essential forage for bees across locations and seasons (Lowenstein et al. 2019; Turo et al. 2021).

**TABLE 1:** Nectar metal concentrations by floral species.

Flower species						
Species		Arsenic	Cadmium	Chromium	Lead	Sum metals
*Asclepias* spp.	Mean ± SE	5.01 ± 1.74^a^	0.01 ± 0.005^ab^	8.19 ± 5.39^ab^	12.8 ± 3.42	25.99 ± 5.54
	Min—max	2.79–8.44	0–0.02	1.79–18.91	8.17–19.46	
*Cichorium intybus*	Mean ± SE	0.70 ± 0.40^bc^	9.31 ± 4.27^ab^	15.6 ± 6.71^a^	35.1 ± 9.88	60.71 ± 13.28
	Min—max	0–2.32	0–32.99	2.51–45.69	6.54–82.49	
*Convolvulus arvensis*	Mean ± SE	*b.d* ^bc^	*b.d* ^ab^	6.12 ± 4.21^ab^	26.1 ± 6.45	32.22 ± 7.51
	Min—max	0	0	1.60–14.52	14.98–37.31	
*Daucus carota*	Mean ± SE	2.99 ± 0.99^ab^	12.5 ± 5.48^a^	7.88 ± 2.42^ab^	15.8 ± 1.67	39.26 ± 5.66
	Min—max	1.51–5.94	1.74–27.78	2.52–13.44	14.60–19.01	
*Lotus corniculatus*	Mean ± SE	0.76 ± 0.76^bc^	0.11 ± 0.11^b^	6.04 ± 5.99^ab^	12.2 ± 9.33	19.06 ± 15.17
	Min—max	0–3.82	0–0.56	0–0.18	0–48.75	
*Securigera varia*	Mean ± SE	0.25 ± 0.16^c^	1.41 ± 0.39^ab^	0.51 ± 0.16^ab^	5.72 ± 0.97^a^	7.90 ± 1.00
	Min—max	0–0.82	0.40–2.81	0–1.03	2.32–7.60	
*Taraxacum*	Mean ± SE	1.78 ± 1.78^abc^	1.70 ± 0.80^ab^	2.99 ± 2.45^ab^	19.4 ± 16.1	25.92 ± 21.13
	Min—max	0–3.56	0.91–2.5	0.54–5.46	3.35–35.55	
*Trifolium pratense*	Mean ± SE	0.57 ± 0.13^c^	1.78 ± 0.27^b^	1.78 ± 0.53^b^	14.9 ± 1.79	18.98 ± 2.25
	Min—max	0–1.35	0.31–3.75	0.01–8.02	6.62–28.99	
*Trifolium repens*	Mean ± SE	0.38 ± 0.32^c^	2.79 ± 1.72^ab^	1.28 ± 0.65^b^	38.5 ± 12.1^a^	42.92 ± 12.50
	Min—max	0–3.26	0–17.81	0–5.11	7.87–135.31	
Average		1.38^a^	3.29^a^	5.59^a^	20.05^b^	

*Note:* Each value represents the average concentrations detected in each flower species combined for all neighborhoods. Values represent the mean concentrations in μg/L ± SE. Min—max represents the highest and lowest measured metal concentration for each variable. ^a,b,c^—Different superscript letters in the same column indicate significantly different values of metals between different flower species (Tukey's honest significant difference test, *p < 0.05*).

Abbreviation: *b.d*, below detection.

Plant species varied in their uptake of individual metals, with each species taking up different concentrations of metals within the same site. Plants have three basic strategies in response to metals: avoidance, detoxification, and biochemical tolerance (Shaw 1989), which dictate the concentrations of metals that are taken up by each species. Generally, lead was found in the highest concentration within nectar, followed by chromium, cadmium, then arsenic across plant species. Only *T. officinale* did not differ significantly in the quantity of each metal found in nectar, suggesting this species may have an ability to limit metal uptake into nectar compared to other floral species present at the same locations. Individually, *A. syriaca* *L*. accumulated the highest concentrations of arsenic compared to the other test species, *D. carota* accumulated the highest concentration of cadmium, *C. intybus* accumulated the highest concentration of chromium, and *T. repens* accumulated the most lead within its nectar (Table 1). Plant species vary widely in their capacity to uptake (Liu et al. 2010) and translocate metals within tissues (Huang and Cunningham 1996) with uptake depending on environmental factors such as pH, temperature, moisture, and organic matter, and physiological characteristics such as root systems and mechanisms of transportation and detoxification (An et al. 2011). Therefore, it is critical to determine the ability of pollinator‐attractive plants to uptake metals prior to establishment within contaminated areas and to manage volunteer species that are tolerant of metals and may pose an exposure risk to bees and other wildlife.

**FIGURE 3:**
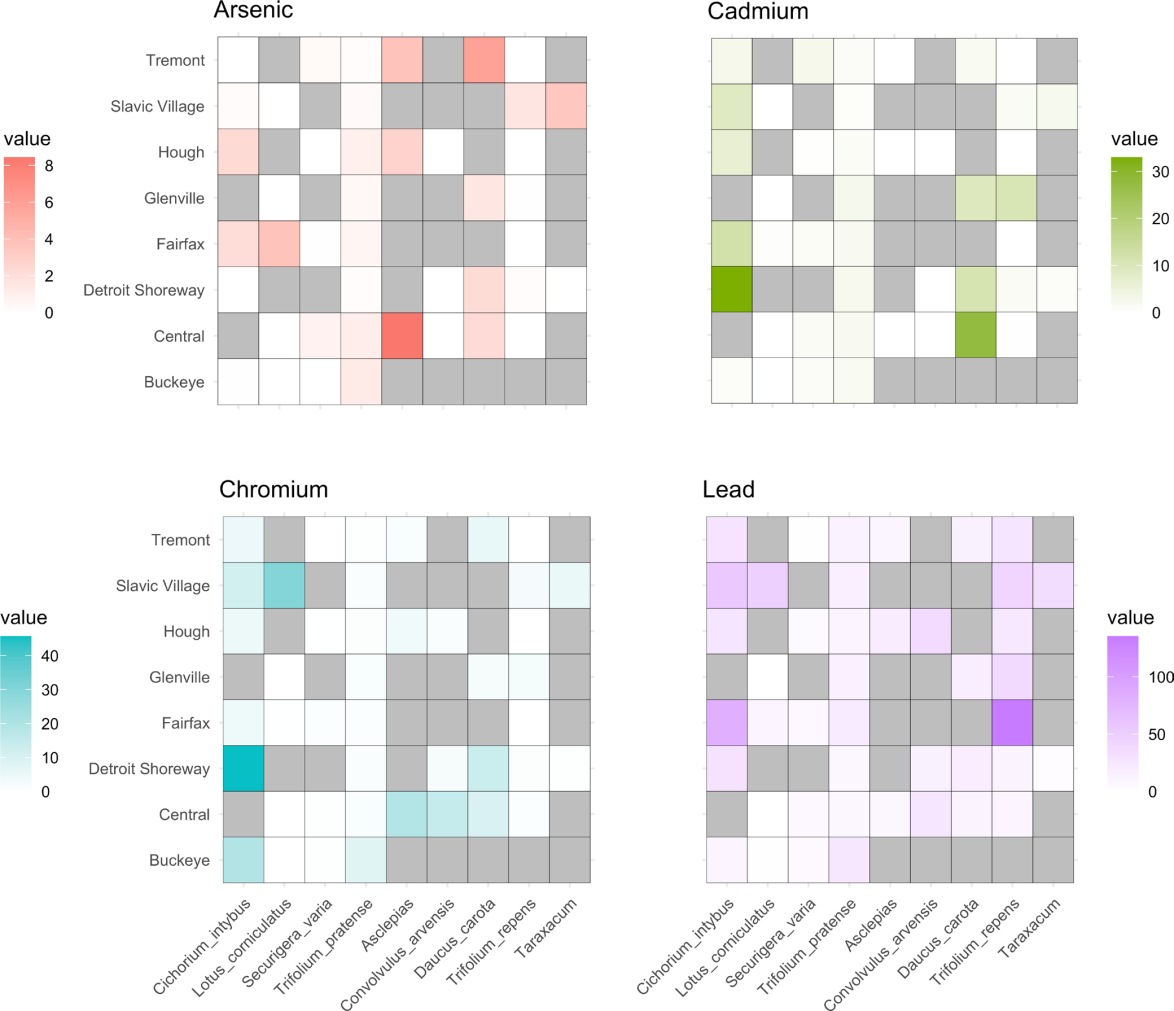
Heatmaps of the average metal concentrations for each floral species by location for individual metals. Values represent the average value of metal measured in nectar from each floral species at each location. The darker color square represents a higher value, the lighter the square the lower the value. Gray squares represent missing values–i.e., a plant species was not present at the sample location.

Among the vacant lots sampled, we found that total nectar metal concentrations varied significantly for some, but not all, flower species. Total nectar metal concentrations varied significantly between sampled vacant lots for *L. corniculatus* (*χ* ^2^ (4) = 10.05, *p* = 0.03) and *T. officianale* (*χ* ^2^ (1) = 4.08, *p* = 0.04), but did not vary significantly between locations for the other species. However, individual metals (As, Cd, Cr, or Pb) did not vary significantly between locations for *L. corniculatus* , *T. officianale*, or any other species (see Figure 3). This study is limited by the absence of some floral species in some of the vacant lots sampled, which complicates the interpretation of whether observed patterns are influenced by species‐specific trends, local environmental conditions, or interactions between these factors. Potential factors impacting flower presence per vacant lot include the frequency of management practices such as mowing, which may limit flowering ability by controlling plant height; the presence of metals at concentrations that inhibit species establishment and success; the influence of other chemicals on plant community structure; or other unknown variables.

### 3.2 Nectar Metals by Location

**FIGURE 4:**
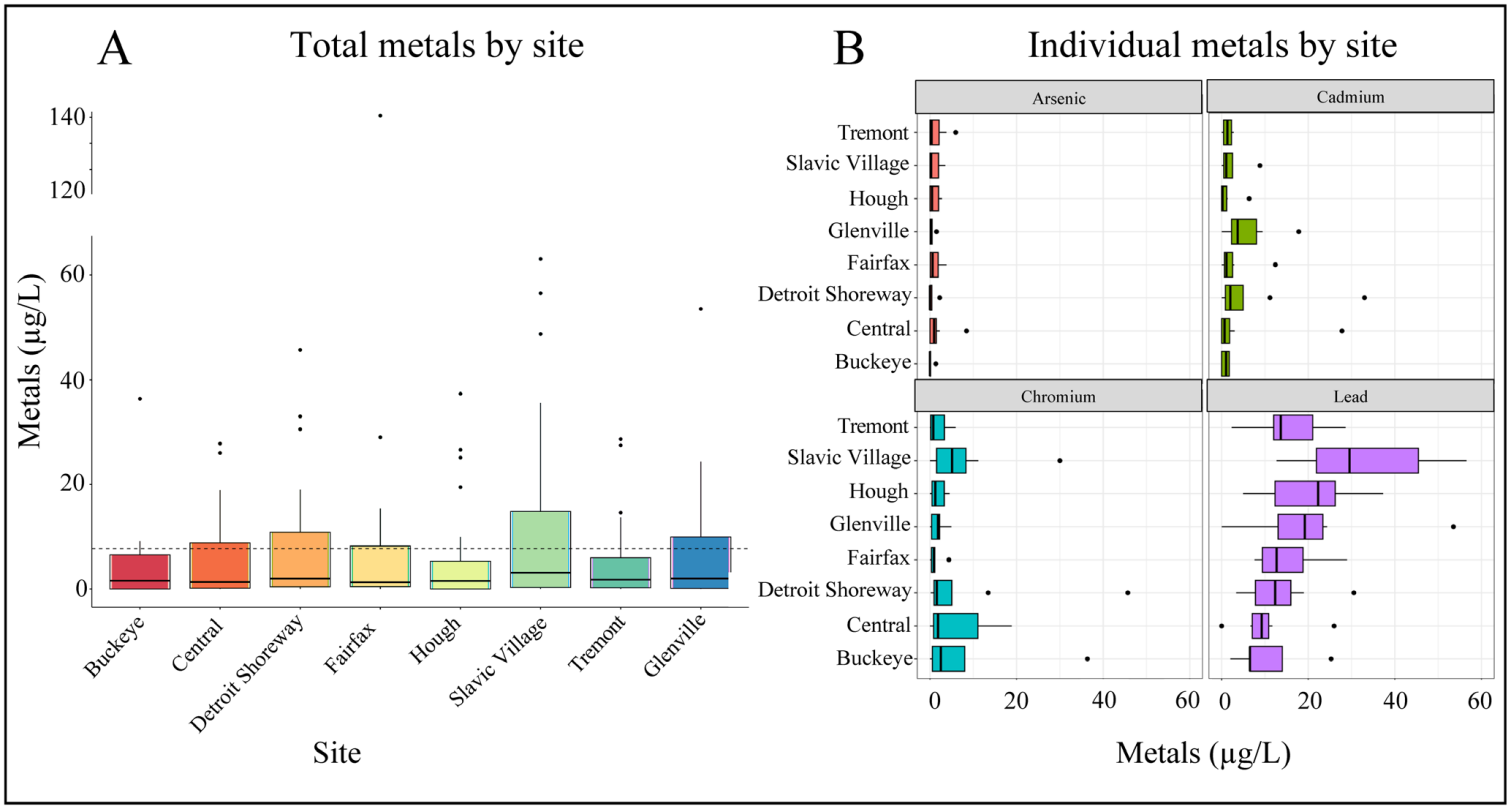
Metals by location. (A) Total nectar metal (measured arsenic, cadmium, chromium, and lead) from all floral species collected combined per site. The dotted line represents the average concentration of metals measured across all sites. (B) Individual nectar metals per site. Each graph represents the total metals from all floral species from each site combined.

Soil metal species and concentrations often vary at small scales within habitat patches, making trace metal distributions difficult to predict. Total nectar metal concentrations for all test metals combined and individual metals did not differ significantly between vacant lot sites. As all floral species tested were not present at every location, some of the results may be attributed to the presence or absence of certain species at each site. However, individual metal concentrations in nectar varied significantly within each site, with each location having higher lead levels than arsenic (*z* = 9.19, *p* < 0.01), cadmium (*z* = 6.73, *p* < 0.01), and chromium (*z* = 5.84, *p* < 0.01) (Table 2, Figure 4). Metal concentrations within individual vacant lots and across all sites studied followed the trend: Pb > Cr > Cd > As. This nectar metal trend follows soil metal concentration trends, as measured by Sharma et al. (2015a, 2015b), see also Figure S1. Cleveland, OH, vacant lot soils have the highest lead concentrations, followed by chromium, arsenic, then cadmium (Sharma et al. 2015a). Although we did not measure soil metal content at each nectar collection study site, exploring the link between soil and nectar metal concentrations could help identify areas where soil conditions predict elevated nectar metal levels.

**TABLE 2:** Nectar metal concentrations by location. Each value represents the average concentrations detected in all flower species combined for each neighborhood. Values represent the mean concentrations in μg/L ± SE. Min – max represents the highest and lowest measured metal concentrations for each variable.

Neighborhood						
Location		Arsenic	Cadmium	Chromium	Lead	Sum metals
Buckeye	Mean ± SE	0.29 ± 0.26	0.92 ± 0.40	9.49 ± 6.86	10.87 ± 4.09	21.57
	Min—max	0–1.35	0–1.83	0–36.35	2.02–25.29	
Central	Mean ± SE	0.76 ± 0.32	4.79 ± 3.85	4.23 ± 2.14	10.35 ± 2.99	20.13
	Min—max	0–2.28	0–27.80	0–14.52	0–25.98	
Detroit Shoreway	Mean ± SE	0.38 ± 0.27	6.59 ± 3.97	8.28 ± 5.55	13.51 ± 2.98	28.76
	Min—max	0–2.25	0–32.98	0.16–45.69	3.35–30.53	
Fairfax	Mean ± SE	1.20 ± 0.62	3.02 ± 1.92	1.25 ± 0.65	46.63 ± 21.05	52.1
	Min—max	0–3.82	0–12.41	0–4.37	7.6–135.31	
Glenville	Mean ± SE	0.43 ± 0.23	6.08 ± 2.67	1.82 ± 0.75	21.28 ± 7.33	29.61
	Min—max	0–1.51	0–17.81	0–4.96	0–53.53	
Hough	Mean ± SE	1.01 ± 0.51	1.36 ± 1.02	1.86 ± 0.77	20.56 ± 4.81	24.79
	Min—max	0–2.79	0–6.36	0–4.54	4.95–37.32	
Slavic Village	Mean ± SE	1.11 ± 0.60	2.27 ± 1.16	7.82 ± 3.96	37.35 ± 19.23	48.55
	Min—max	0–3.56	0–8.85	0–30.00	12.66–63.07	
Tremont	Mean ± SE	1.50 ± 0.90	1.37 ± 0.44	1.96 ± 0.92	15.82 ± 3.52	20.65
	Min—max	0–3.81	0–2.81	0–4.86	2.32–28.65	
Average		0.84	3.30	4.59	22.05	

**FIGURE 5:**
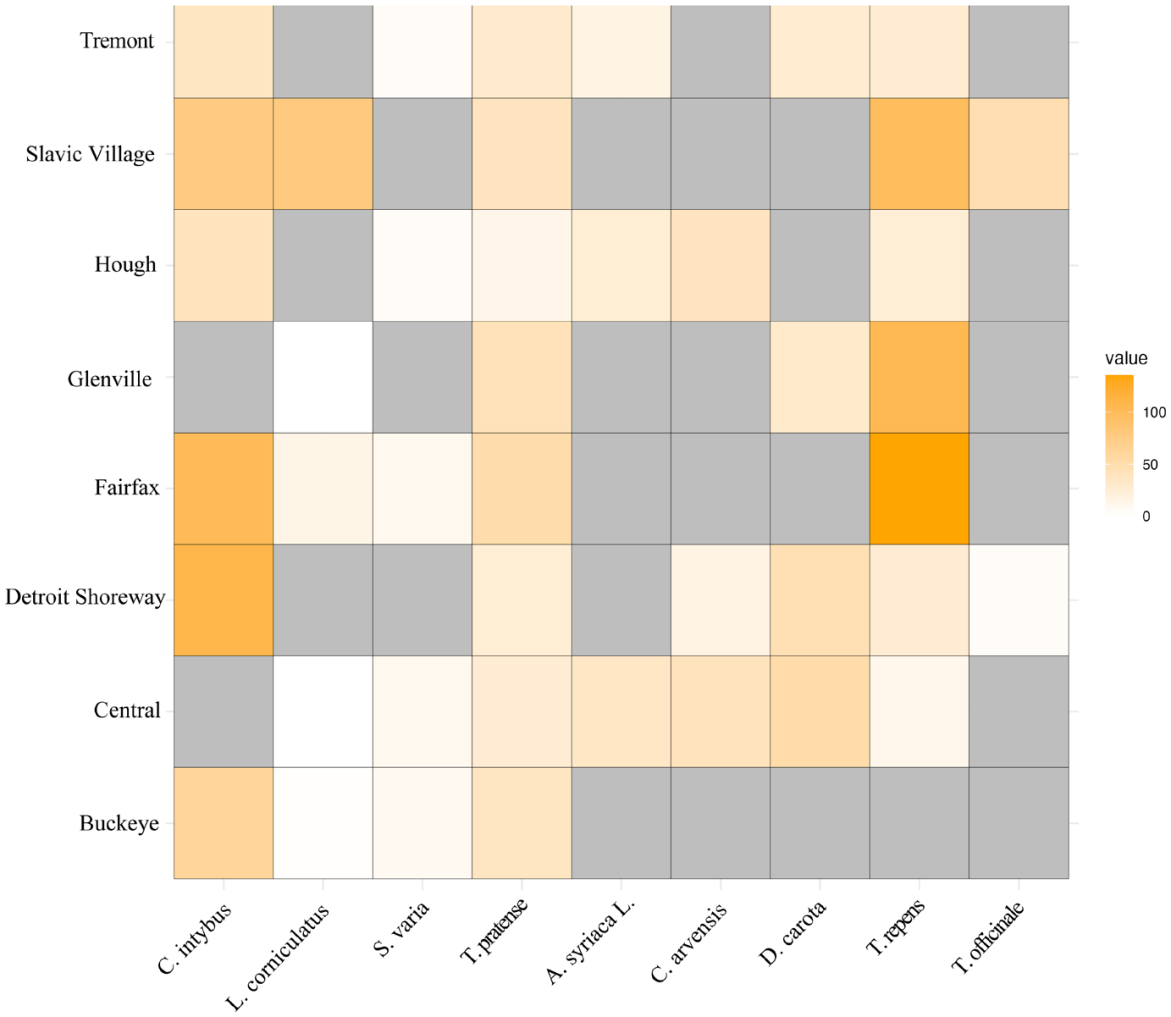
Heatmap of the sum of all metals by floral species and location. Values represent the sum of all metals (arsenic, cadmium, chromium, lead) for each flower species per location. The darker color square represents a higher value. Gray squares represent missing values–i.e., a plant species was not present at the sample location.

Nectar metal concentrations varied significantly by plant species within the same vacant lot at two locations: Central (*χ* ^2^ (6) = 13.03, *p* = 0.04) and Glenville (*χ* ^2^ (3) = 8.12, *p* = 0.04), but did not vary significantly between species in other vacant lots, (Figure 5). Within the Central neighborhood vacant lot, *D. carota* accumulated significantly higher nectar metals than *L. corniculatus* (*z* = 3.29, *p* = 0.01), and within the Glenville neighborhood vacant lot, *T. repens* (*z* = 2.47, *p* = 0.02), *T. pratense* (*z* = 2.39, *p* = 0.02), and *D. carota* (*z* = 2.47, *p* = 0.01) all had higher nectar metal loads than *L. corniculatus* .

Contemporary airborne lead particulate may explain the observed trends in nectar lead concentrations. As lead has low solubility and readily sorbs with soil, it is typically concentrated in the roots of plants (Hladun et al. 2015). Lead uptake into nectar has been documented in certain species such as *Cucurbita pepo* (Xun et al. 2017), but generally, has lower translocation ability than other metals, such as cadmium or zinc. Airborne lead particles from soil disturbances such as demolition or construction (Jacobs et al. 2013) may have settled on floral surfaces and been incorporated into nectar during extraction. For instance, from 2010 to 2019 approximately 8000 homes were demolished in Cleveland (Cuyahoga Land Bank, 2019), many of which were built prior to legislation outlawing the use of lead paint sales (“40 CFR Part 745—Lead‐Based Paint Poisoning Prevention in Certain Residential Structures n.d.”).

Many factors must be considered when managing vacant land for pollinators (Turo and Gardiner 2020), including the potential need to mitigate pollutants. Management efforts that focus solely on providing forage may be insufficient if nectar resources remain contaminated, as this can lead to suboptimal foraging behavior and reduced pollinator fitness (Moroń et al. 2012; Sivakoff et al. 2020). Furthermore, the presence of metals in nectar can alter pollinator preferences (Meindl and Ashman 2013; Burden et al. 2019), potentially reshaping plant‐pollinator networks. As certain metals can bioaccumulate in bumble bees life stages (Scott et al. 2024), even low nectar metal levels could have detrimental long‐term effects. Although measured nectar metal concentrations in this study were much lower than lethal levels, pollinators visit hundreds of flowers throughout their lifespan. Combined with the potential for bioaccumulation, this could lead to the buildup of metals to potentially harmful levels.

## 4 Conclusion

The presence of trace metals in nectar provides a direct exposure pathway for nectar‐feeding animals to these contaminants. Metals were detected in the nectar of all flowering test species, confirming oral consumption as a significant route of exposure. Given that these floral resources are essential forage for urban pollinators (Lowenstein et al. 2019), coupled with the potential for certain metals to bioaccumulate in bee bodies, there is a clear risk of metal toxicity. This research underscores the need to consider soil metal chemistry, alongside pesticides and other stressors, when selecting locations for habitat initiatives, such as vacant lots or urban gardens. To improve the success of conservation programs, it is vital to incorporate strategies for monitoring environmental contamination and remediating polluted habitats during the planning stages of habitat establishment. Proactively managing floral habitats within known contaminated landscapes –through interventions such as mowing prior to bloom or planting species that limit metal uptake into nectar –can mitigate the risk of pollinators foraging on contaminated resources. Addressing anthropogenic sources of metal pollution, including improper waste disposal and chemical residues, alongside public policies that promote sustainable land use, will ensure that restored habitats provide not only abundant floral resources but also safe foraging environments for pollinators.

## Author Contributions

**Sarah B. Scott:** conceptualization (equal), data curation (lead), formal analysis (lead), investigation (lead), methodology (lead), visualization (lead), writing – original draft (lead), writing – review and editing (equal). **Mary M. Gardiner:** conceptualization (equal), funding acquisition (equal), supervision (lead), writing – review and editing (equal).

## Conflicts of Interest

The authors declare no conflicts of interest.

## Supporting information


**Figure S1.** Soil metal concentrations from neighborhoods in Cleveland, OH vacant lots.

## Data Availability

The data that support the findings of this study are openly available in Dryad at DOI: 10.5061/dryad.rbnzs7hnj

## References

[ece371238-bib-0001] n.d. “40 CFR Part 745—Lead‐Based Paint Poisoning Prevention in Certain Residential Structures.” Accessed March 5, 2025. https://www.ecfr.gov/current/title‐40/part‐745.

[ece371238-bib-0002] An, L. , Y. Pan , Z. Wang , and C. Zhu . 2011. “Heavy Metal Absorption Status of Five Plant Species in Monoculture and Intercropping.” Plant and Soil 345: 237–245.

[ece371238-bib-0003] Anderson, E. C. , and E. S. Minor . 2017. “Vacant Lots: An Underexplored Resource for Ecological and Social Benefits in Cities.” Urban Forestry & Urban Greening 21: 146–152.

[ece371238-bib-0004] Arnold, P. M. , and H. J. Michaels . 2017. “Nectar Sampling for Prairie and Oak Savanna Butterfly Restoration.” Applications in Plant Sciences 5, no. 6: 1600148. 10.3732/apps.1600148. PMC5499304 28690931

[ece371238-bib-0005] Aronson, M. F. J. , C. H. Nilon , C. A. Lepczyk , et al. 2016. “Hierarchical Filters Determine Community Assembly of Urban Species Pools.” Ecology 97: 2952–2963. 27870023 10.1002/ecy.1535

[ece371238-bib-0006] Belsky, J. , and N. K. Joshi . 2020. “Effects of Fungicide and Herbicide Chemical Exposure on Apis and Non‐Apis Bees in Agricultural Landscape.” Frontiers in Environmental Science 8: 81. 10.3389/fenvs.2020.00081.

[ece371238-bib-0007] Breidenbach, L. R. , L. Benner , M. Roß‐Nickoll , V. Linnemann , and A. Schäffer . 2023. “Monitoring Metal Patterns From Urban and Agrarian Sites Using the Bumblebee *Bombus terrestris* as a Bioindicator.” Environmental Science and Pollution Research 30, no. 57: 119947–119960. 10.1007/s11356-023-30504-w. 37934407 PMC10698117

[ece371238-bib-0008] Brown, M. J. F. , M. J. Brown , L. V. Dicks , et al. 2016. “A Horizon Scan of Future Threats and Opportunities for Pollinators and Pollination.” PeerJ 4: e2249. 10.7717/peerj.2249. 27602260 PMC4991895

[ece371238-bib-0009] Burden, C. M. , M. O. Morgan , K. R. Hladun , G. V. Amdam , J. J. Trumble , and B. H. Smith . 2019. “Acute Sublethal Exposure to Toxic Heavy Metals Alters Honey Bee (*Apis mellifera*) Feeding Behavior.” Scientific Reports 9: 1–10. 30862878 10.1038/s41598-019-40396-x PMC6414635

[ece371238-bib-0010] Cheng, Z. , A. Paltseva , I. Li , et al. 2015. “Trace Metal Contamination in new York City Garden Soils.” Soil Science 180, no. 4: 167–174. 10.1097/ss.0000000000000126.

[ece371238-bib-0011] Chojnacka, K. , A. Chojnacki , H. Górecka , and H. Górecki . 2005. “Bioavailability of Heavy Metals From Polluted Soils to Plants.” Science of the Total Environment 337, no. 1: 175–182. 10.1016/j.scitotenv.2004.06.009. 15626388

[ece371238-bib-0012] Exner, R. 2019. “Cleveland's Population Flattens Near 385,000 After Decades of Big Losses, New Census Estimates Say.” https://www.cleveland.com/news/g66l‐2019/05/d1695a54c89135/clevelands‐population‐flattens‐near‐385000‐after‐decades‐of‐big‐losses‐new‐census‐estimates‐say.html.

[ece371238-bib-0013] Gao, S. , F. Zheng , L. Yue , and B. Chen . 2024. “Chronic Cadmium Exposure Impairs Flight Behavior by Dampening Flight Muscle Carbon Metabolism in Bumblebees.” Journal of Hazardous Materials 466: 133628. 38301442 10.1016/j.jhazmat.2024.133628

[ece371238-bib-0014] Gibson, C. , and United States Census Bureau . 1998. “Population of the 100 Largest Cities and Other Urban Places In The United States: 1790 to 1990. Working Paper POP‐WP027.” https://www.census.gov/library/working‐papers/1998/demo/POP‐twps0027.html.

[ece371238-bib-0015] Gradish, A. E. , J. van der Steen , C. D. Scott‐Dupree , et al. 2019. “Comparison of Pesticide Exposure in Honey Bees (Hymenoptera: Apidae) and Bumble Bees (Hymenoptera: Apidae): Implications for Risk Assessments.” Environmental Entomology 48: 12–21. 30508078 10.1093/ee/nvy168 PMC8215506

[ece371238-bib-0016] Hall, D. M. , G. R. Camilo , R. K. Tonietto , et al. 2017. “The City as a Refuge for Insect Pollinators.” Conservation Biology 31: 24–29. 27624925 10.1111/cobi.12840

[ece371238-bib-0017] Harrison, T. , and R. Winfree . 2015. “Urban Drivers of Plant‐Pollinator Interactions.” Functional Ecology 29: 879–888.

[ece371238-bib-0018] Herrmann, D. L. , W. D. Shuster , A. L. Mayer , and A. S. Garmestani . 2016. “Sustainability for Shrinking Cities.” Sustainability 8: 911.

[ece371238-bib-0019] Hladun, K. R. , D. R. Parker , and J. T. Trumble . 2015. “Cadmium, Copper, and Lead Accumulation and Bioconcentration in the Vegetative and Reproductive Organs of *Raphanus sativus*: Implications for Plant Performance and Pollination.” Journal of Chemical Ecology 41: 386–395. 10.1007/s10886-015-0569-7. 25845355

[ece371238-bib-0020] Huang, J. W. , and S. D. Cunningham . 1996. “Lead Phytoextraction: Species Variation in Lead Uptake and Translocation.” New Phytologist 134: 75–84. 10.1111/j.1469-8137.1996.tb01147.x.

[ece371238-bib-0021] Ecology and Hydrology , D. Spurgeon , H. Hesketh , et al. 2016. “Chronic Oral Lethal and Sub‐Lethal Toxicities of Different Binary Mixtures of Pesticides and Contaminants in Bees ( *Apis mellifera* , Osmia Bicornis and *Bombus terrestris* ).” EFSA Supporting Publications 13: 1076E.

[ece371238-bib-0022] Jacobs, D. E. , S. Cali , A. Welch , et al. 2013. “Lead and Other Heavy Metals in Dust Fall From Single‐Family Housing Demolition.” Public Health Reports 128: 454–462. 24179257 10.1177/003335491312800605 PMC3804089

[ece371238-bib-0023] Kopit, A. M. , and T. L. Pitts‐Singer . 2018. “Routes of Pesticide Exposure in Solitary, Cavity‐Nesting Bees.” Environmental Entomology 47: 499–510.

[ece371238-bib-0024] Kuppler, J. , U. Neumüller , A. V. Mayr , et al. 2023. “Favourite Plants of Wild Bees.” Agriculture, Ecosystems & Environment 342: 108266. 10.1016/j.agee.2022.108266.

[ece371238-bib-0025] Liu, J.‐G. , G.‐H. Li , W.‐C. Shao , J.‐K. Xu , and D.‐K. Wang . 2010. “Variations in Uptake and Translocation of Copper, Chromium and Nickel Among Nineteen Wetland Plant Species.” Pedosphere 20, no. 1: 96–103. 10.1016/S1002-0160(09)60288-5.

[ece371238-bib-0026] Lowenstein, D. M. , K. C. Matteson , and E. S. Minor . 2019. “Evaluating the Dependence of Urban Pollinators on Ornamental, Non‐Native, and ‘Weedy’ Floral Resources.” Urban Ecosystems 22: 293–302.

[ece371238-bib-0027] Martins, K. T. , A. Gonzalez , and M. J. Lechowicz . 2017. “Patterns of Pollinator Turnover and Increasing Diversity Associated With Urban Habitats.” Urban Ecosystems 20, no. 6: 1359–1371. 10.1007/s11252-017-0688-8.

[ece371238-bib-0028] McFrederick, Q. S. , and G. LeBuhn . 2006. “Are Urban Parks Refuges for Bumble Bees Bombus Spp.(Hymenoptera: Apidae)?” Biological Conservation 129: 372–382.

[ece371238-bib-0029] McKinney, M. L. 2002. “Urbanization, Biodiversity, and Conservation: The Impacts of Urbanization on Native Species Are Poorly Studied, but Educating a Highly Urbanized Human Population About These Impacts Can Greatly Improve Species Conservation in all Ecosystems.” Bioscience 52: 883–890.

[ece371238-bib-0030] Meindl, G. A. , and T.‐L. Ashman . 2013. “The Effects of Aluminum and Nickel in Nectar on the Foraging Behavior of Bumblebees.” Environmental Pollution 177: 78–81. 10.1016/j.envpol.2013.02.017. 23466735

[ece371238-bib-0031] Monchanin, C. , A. Blanc‐Brude , E. Drujont , et al. 2021a. “Chronic Exposure to Trace Lead Impairs Honey Bee Learning.” Ecotoxicology and Environmental Safety 212: 112008. 33578129 10.1016/j.ecoenv.2021.112008

[ece371238-bib-0032] Monchanin, C. , E. Drujont , J.‐M. Devaud , M. Lihoreau , and A. B. Barron . 2021b. “Metal Pollutants Have Additive Negative Effects on Honey Bee Cognition.” Journal of Experimental Biology 224: jeb241869. 34002230 10.1242/jeb.241869

[ece371238-bib-0033] Monchanin, C. , E. Drujont , G. Le Roux , et al. 2024. “Environmental Exposure to Metallic Pollution Impairs Honey Bee Brain Development and Cognition.” Journal of Hazardous Materials 465: 133218. 38113738 10.1016/j.jhazmat.2023.133218

[ece371238-bib-0034] Moroń, D. , I. M. Grześ , P. Skórka , et al. 2012. “Abundance and Diversity of Wild Bees Along Gradients of Heavy Metal Pollution.” Journal of Applied Ecology 49: 118–125.

[ece371238-bib-0035] Moroń, D. , H. Szentgyörgyi , P. Skórka , S. G. Potts , and M. Woyciechowski . 2014. “Survival, Reproduction and Population Growth of the Bee Pollinator, *Osmia rufa* (Hymenoptera: Megachilidae), Along Gradients of Heavy Metal Pollution.” Insect Conservation and Diversity 7: 113–121.

[ece371238-bib-0036] Neil, K. , and J. Wu . 2006. “Effects of Urbanization on Plant Flowering Phenology: A Review.” Urban Ecosystems 9: 243–257.

[ece371238-bib-0037] Pecina, V. , D. Juřička , M. Vašinová Galiová , J. Kynický , L. Baláková , and M. Brtnický . 2021. “Polluted Brownfield Site Converted Into a Public Urban Park: A Place Providing Ecosystem Services or a Hidden Health Threat?” Journal of Environmental Management 291: 112669. 33934019 10.1016/j.jenvman.2021.112669

[ece371238-bib-0038] Rascio, N. , and F. Navari‐Izzo . 2011. “Heavy Metal Hyperaccumulating Plants: How and Why Do They Do It? And What Makes Them So Interesting?” Plant Science: An International Journal of Experimental Plant Biology 180: 169–181. 21421358 10.1016/j.plantsci.2010.08.016

[ece371238-bib-0039] Reeves, R. D. , A. J. M. Baker , T. Jaffré , P. D. Erskine , G. Echevarria , and A. van der Ent . 2018. “A Global Database for Plants That Hyperaccumulate Metal and Metalloid Trace Elements.” New Phytologist 218, no. 2: 407–411. 10.1111/nph.14907. 29139134

[ece371238-bib-0040] Rothman, J. A. , L. Leger , J. S. Kirkwood , and Q. S. McFrederick . 2019. “Cadmium and Selenate Exposure Affects the Honey Bee Microbiome and Metabolome, and Bee‐Associated Bacteria Show Potential for Bioaccumulation.” Applied and Environmental Microbiology 85: e01411‐19. 31471302 10.1128/AEM.01411-19 PMC6803295

[ece371238-bib-0041] RStudio Team . 2020. “RStudio|Open Source & Professional Software for Data Science Teams.” https://www.rstudio.com/.

[ece371238-bib-0042] Sampson, N. , J. Nassauer , A. Schulz , K. Hurd , C. Dorman , and K. Ligon . 2017. “Landscape Care of Urban Vacant Properties and Implications for Health and Safety: Lessons From Photovoice.” Health & Place 46: 219–228. 28570993 10.1016/j.healthplace.2017.05.017

[ece371238-bib-0043] Scott, S. , F. Sivakoff , M. Meuti , and M. Gardiner . 2023. “Metals Could Challenge Pollinator Conservation in Legacy Cities.” Journal of Insect Conservation 27: 1–15.

[ece371238-bib-0044] Scott, S. B. , R. Lanno , and M. M. Gardiner . 2024. “Acute Toxicity and Bioaccumulation of Common Urban Metals in *Bombus impatiens* Life Stages.” Science of the Total Environment 915: 169997. 38218493 10.1016/j.scitotenv.2024.169997

[ece371238-bib-0045] Scott, S. B. , F. S. Sivakoff , and M. M. Gardiner . 2022. “Exposure to Urban Heavy Metal Contamination Diminishes Bumble Bee Colony Growth.” Urban Ecosystems 25, no. 3: 989–997. 10.1007/s11252-022-01206-x.

[ece371238-bib-0046] Senapathi, D. , M. A. Goddard , W. E. Kunin , and K. C. R. Baldock . 2017. “Landscape Impacts on Pollinator Communities in Temperate Systems: Evidence and Knowledge Gaps.” Functional Ecology 31: 26–37.

[ece371238-bib-0047] Sharma, K. , N. T. Basta , and P. S. Grewal . 2015a. “Soil Heavy Metal Contamination in Residential Neighborhoods in Post‐Industrial Cities and Its Potential Human Exposure Risk.” Urban Ecosystems 18: 115–132.

[ece371238-bib-0048] Sharma, K. , Z. Cheng , and P. S. Grewal . 2015b. “Relationship Between Soil Heavy Metal Contamination and Soil Food Web Health in Vacant Lots Slated for Urban Agriculture in Two Post‐Industrial Cities.” Urban Ecosystems 18: 835–855.

[ece371238-bib-0049] Shaw, J. 1989. Heavy Metal Tolerance in Plants: Evolutionary Aspects. CRC Press.

[ece371238-bib-0050] Sivakoff, F. S. , S. P. Prajzner , and M. M. Gardiner . 2020. “Urban Heavy Metal Contamination Limits Bumblebee Colony Growth.” Journal of Applied Ecology 57, no. 8: 1561–1569. 10.1111/1365-2664.

[ece371238-bib-0051] Theodorou, P. , R. Radzevičiūtė , G. Lentendu , et al. 2020. “Urban Areas as Hotspots for Bees and Pollination but Not a Panacea for All Insects.” Nature Communications 11, no. 1: 576. 10.1038/s41467-020-14496-6. PMC6989530 31996690

[ece371238-bib-0052] Turo, K. J. , and M. M. Gardiner . 2020. “The Balancing Act of Urban Conservation.” Nature Communications 11: 3773. 10.1038/s41467-020-17539-0 PMC7391721 32728191

[ece371238-bib-0053] Turo, K. J. , M. R. Spring , F. S. Sivakoff , Y. A. D. Flor , and M. M. Gardiner . 2021. “Conservation in Post‐Industrial Cities: How Does Vacant Land Management and Landscape Configuration Influence Urban Bees?” Journal of Applied Ecology 58, no. 1: 58–69. 10.1111/1365-2664.13773.

[ece371238-bib-0054] Vaudo, A. D. , J. F. Tooker , C. M. Grozinger , and H. M. Patch . 2015. “Bee Nutrition and Floral Resource Restoration.” Current Opinion in Insect Science 10: 133–141. 29588000 10.1016/j.cois.2015.05.008

[ece371238-bib-0055] Western Reserve land Conservancy . 2023. “2023 Cleveland Property Inventory.” https://wrlandconservancy.org/neighborhood‐stabilization/property‐inventories/cleveland‐property‐inventory‐2023/.

[ece371238-bib-0056] Wu, L. , Q. Sun , J. Zhao , X. Wang , D. Wang , and Y. Zhang . 2025. “Effects of Heavy Metal Accumulation Mediated by Floral Rewards on Key Stages of Growth and Development of Bumblebees (*Bombus terrestris* L.).” Environmental Toxicology and Chemistry 44, no. 1: vgae035. 10.1093/etojnl/vgae035. 39887284

[ece371238-bib-0057] Xun, E. , Y. Zhang , J. Zhao , and J. Guo . 2017. “Translocation of Heavy Metals From Soils Into Floral Organs and Rewards of *Cucurbita pepo* : Implications for Plant Reproductive Fitness.” Ecotoxicology and Environmental Safety 145: 235–243. 28738207 10.1016/j.ecoenv.2017.07.045

[ece371238-bib-0058] Zioga, E. , B. White , and J. C. Stout . 2023. “Pesticide Mixtures Detected in Crop and Non‐Target Wild Plant Pollen and Nectar.” Science of the Total Environment 879: 162971. 36958551 10.1016/j.scitotenv.2023.162971

