# Supplementary figures and images for: Trace Metals in Nectar of Important Urban Pollinator Forage Plants: A Direct Exposure Risk to Pollinators and Nectar‐Feeding Animals in Cities
Source: Ecol Evol. 2025 Apr 15;15(4):e71238. doi: 10.1002/ece3.71238 (PMC11999714; doi:10.1002/ece3.71238)

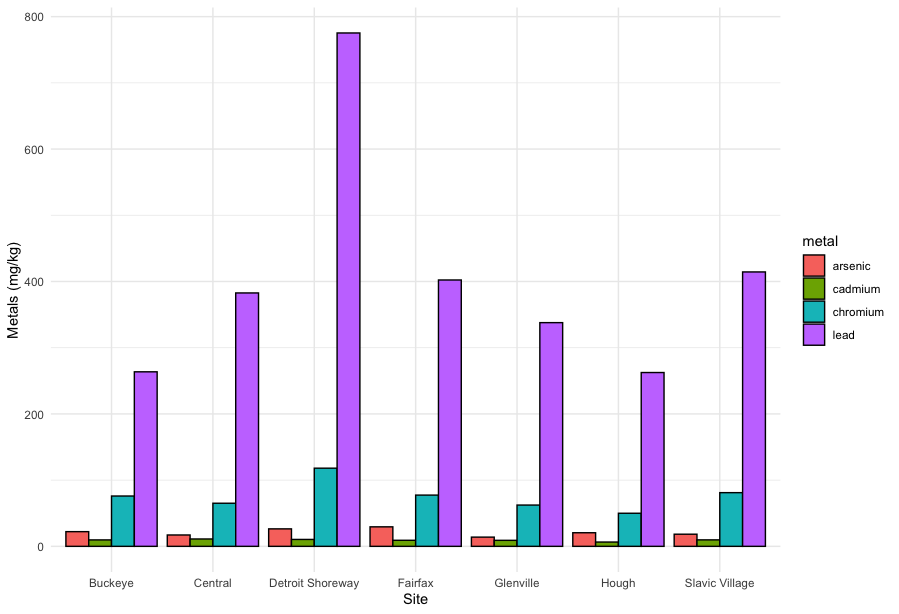

Supplement: Supplementary file 1 — Figure S1. Soil metal concentrations from neighborhoods in Cleveland, OH vacant lots. [file ECE3-15-e71238-s001.png]
